# Mapping the Voxel-Wise Effective Connectome in Resting State fMRI

**DOI:** 10.1371/journal.pone.0073670

**Published:** 2013-09-12

**Authors:** Guo-Rong Wu, Sebastiano Stramaglia, Huafu Chen, Wei Liao, Daniele Marinazzo

**Affiliations:** 1 Faculty of Psychology and Educational Sciences, Department of Data Analysis, Ghent University, Ghent, Belgium; 2 Key Laboratory for NeuroInformation of Ministry of Education, School of Life Science and Technology, University of Electronic Science and Technology of China, Chengdu, China; 3 Dipartimento di Fisica, Università degli Studi di Bari and INFN, Bari, Italy; 4 Center for Cognition and Brain Disorders and the Affiliated Hospital, Hangzhou Normal University, Hangzhou, China; Cuban Neuroscience Center, Cuba

## Abstract

A network approach to brain and dynamics opens new perspectives towards understanding of its function. The functional connectivity from functional MRI recordings in humans is widely explored at large scale, and recently also at the voxel level. The networks of dynamical directed connections are far less investigated, in particular at the voxel level. To reconstruct full brain effective connectivity network and study its topological organization, we present a novel approach to multivariate Granger causality which integrates information theory and the architecture of the dynamical network to efficiently select a limited number of variables. The proposed method aggregates conditional information sets according to community organization, allowing to perform Granger causality analysis avoiding redundancy and overfitting even for high-dimensional and short datasets, such as time series from individual voxels in fMRI. We for the first time depicted the voxel-wise hubs of incoming and outgoing information, called Granger causality density (GCD), as a complement to previous repertoire of functional and anatomical connectomes. Analogies with these networks have been presented in most part of default mode network; while differences suggested differences in the specific measure of centrality. Our findings could open the way to a new description of global organization and information influence of brain function. With this approach is thus feasible to study the architecture of directed networks at the voxel level and individuating hubs by investigation of degree, betweenness and clustering coefficient.

## Introduction

Resting-state functional magnetic resonance imaging (rs-fMRI) is increasingly being used to investigate brain dynamics [1]. The dynamical integration between brain areas, evidencing neuronal communications beyond the underlying anatomical structure, is investigated by functional and effective connectivity. Functional connectivity (FC) measures statistical dependencies of time-series between distinct units; while effective connectivity (EC) investigates the influence one neuronal system exerts over another, by means of predictive models [2]. The former has been comprehensively described and integrated in the functional connectome of the human brain [3]. Nevertheless, only a few studies have investigated the large-scale directed influence brain network based on EC [4], [5], though not yet at the voxel level.

Once that the architecture of a neural network is known, it is possible to identify its functional hubs and critical nodes, determining preferred pathways of neuronal communication and estimating the controllability of a system [6], or to use the graph structure as a decoding tool for brain states [7]. A graph-theoretical approach to whole brain functional connectivity, based on the count of the number of functional connections per voxel (edges in graph) has been successfully applied [8]–[15] allowing to identify the distribution of functional hubs. Prominent functional hubs were identified in the default mode network as well as in dorsal, parietal and prefrontal regions.

A significant advance in the understanding of brain function could come from the investigation of directed networks of information transfer, such as those based on effective connectivity. The models on which effective connectivity is based can either be physiologically motivated, such as dynamical causal models, or purely data-driven such as in Granger causality (GC) analysis (for an extensive review see [16]). GC [17], which evaluates whether the prediction error on one variable is significantly reduced by including another variable in the autoregressive (AR) model, has been used to identify the effective connectivity of blood-oxygen-level-dependent (BOLD) fMRI signals [18]–[20]. It is worth to note that the application of GC to fMRI is controversial [21], [22], especially for resting-state fMRI [16]. Nonetheless, the analogies and differences between network architectures of functional connectivity and GC-based effective connectivity have been investigated [5], [23], [24]. Those studies are based on coarse-grained parcellations from anatomically based brain atlases. Little is known on the functional hubs in voxel-wise EC network. The main issue arising when applying Granger causality to high dimensional networks, such as voxel time series from the whole brain, is the curse of dimensionality in the conditioning variables.

To cope with redundancy and dimensionality issues in evaluating multivariate GC, it has recently been proposed [25] that conditioning on a small number of variables, chosen as the most informative ones for each given driver, can be enough to recover a network of effective connectivity eliminating spurious influences in particular when the connectivity pattern is sparse. We refer to this approach as the partially conditioned GC (PCGC). Another issue related with the recovery of EC networks from BOLD signal is the possibly confounding effect of the hemodynamic response. In order to decouple the neuronal activity and the hemodynamic responses, we applied a blind deconvolution procedure, based on the detection of pseudo-events, to the BOLD signal [26].

## Materials and Methods

### Subjects and Data Acquisition

The resting-state fMRI dataset used in this study has been publicly released under the ‘1000 Functional Connectomes Project’ (http://fcon_1000.projects.nitrc.org, accessed March 2012).and has been collected at the State Key Laboratory of Cognitive Neuroscience and Learning at Beijing Normal University (n = 197, 122 females; age: 21.2±1.8 years). All participants had no history of neurological and psychiatric disorders. Written informed consent was obtained from each participant, and the study was approved by the local Institutional Review Board. During the resting state, participants were instructed to keep still with their eyes closed but not to fall asleep, remaining as motionless as possible. The fMRI images were acquired by using single-shot gradient echo planar imaging (EPI) sequence (repetition time (TR): 2000 ms; echo time: 30 ms; axial slices: 33; thickness: 3 mm; inter-slice gap: 0.6 mm; field of view: 200×200 mm^2^; in-plane resolution: 64×64; flip angle: 90°). For each subject, a total of 225-volumes were acquired, resulting in a total scan time of 450 s.

### Data Preprocessing

Preprocessing of resting-state images was performed using SPM8: data underwent slice timing correction, realigning of all the images to the first image using six degrees of freedom rigid body transformations, spatial normalization into the Montreal Neurological Institute template then resampling to 3-mm isotropic voxels, and spatial smoothing using a 6-mm full-width half-maximum Gaussian kernel. Recently, small head movements have been identified as an important confounding factor for resting state fMRI studies [27]–[29]. To limit the impact of micro-movements artifacts on these data, we implemented a ‘scrubbing’ procedure as part of data preprocessing. An estimate of head motion at each time point was calculated as the frame-wise displacement (FD) (mean absolute FD across all subject = 0.104±0.045 mm), using six displacements from rigid body motion correction procedure mentioned above [27]. Following [30], any image with FD>0.5 mm was removed and replaced by a linear interpolation.

Additional parameters were used to remove possible spurious variances from the data through linear regression. These were 1) six head motion parameters obtained in the realigning step, 2) signal from a region in cerebrospinal fluid, 3) signal from a region centered in the white matter, 4) global signal averaged over the whole brain. Time series were linearly detrended and temporally band-pass filtered (0.01–0.08 Hz). We then generated a study-specific functional volume mask that included only voxels present in all participants.

### Spontaneous Point Event Detection and HRF Deconvolution

Previous studies have shown that the hemodynamic processes are inhomogeneous across the whole brain [31]; in order to maximally eliminate the effect of hemodynamic response which may disturb the inference of temporal precedence [32], we employed a blind deconvolution technique developed for resting-state BOLD-fMRI signal [26], starting from the idea that the resting-state BOLD spikes are due to spontaneous point events, based on the increasing evidence of non-random patterns of BOLD spike that govern the dynamics of the brain at rest [33]–[35]. These spontaneous events can be detected by point process analysis (PPA), picking up BOLD fluctuations of relatively large amplitude [36], [37]. After detecting these resting-state BOLD transients, the neural event onsets are stored for further HRF reconstruction. Voxel-specific HRF is obtained by fitting raw BOLD signal with canonical HRF and its time derivative, in order to finally recover signals at the neural level by Wiener deconvolution (Matlab code is available at http://users.ugent.be/~dmarinaz/code.html) [38].

### Partially Conditioned Granger Causality

Partially conditioned Granger causality (PCGC) was originally proposed in [25] as a technique able to compute the GC conditioned to a small number of variables in the framework of information theory. The idea is that conditioning on a small number of the most informative variables for the candidate driver variable is sufficient to remove indirect interactions especially for sparse connectivity patterns. Here we briefly report the foundations of the approach.

Let’s consider 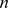 covariance-stationary variables 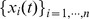; the state vectors, representing the past realizations up to a lag 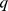 are denoted as 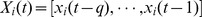.  The multivariate Granger causality from variable 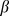 to variable 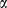 is defined as the logarithm of the ratio of 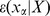, the mean squared error prediction of 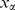 on the basis of all the vectors 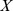, and 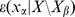, the mean squared error prediction of 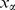 on the basis of the past of all variables but 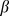. What was proposed is a reduction of the number of variables to be included in the conditioning dataset.

The PCGC index 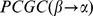
is defined as follows:
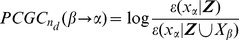
(1)where 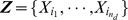
is a set of the 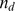 variables, in 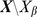, most informative for 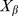.

In order to choose the first variable of the subset, the mutual information between the candidate driver variable and each of the other variables is estimated; the second variable of the subset is selected among the remaining ones, as those that, jointly with the previously chosen variable, maximize the mutual information with the driver variable. Then, one keeps adding the rest of the variables by iterating this procedure. This is repeated until the addition of another variable does not result in a substantial information gain.

The model order for PCGC analysis can be chosen by standard methods such as the Akaike information criterion, the Bayesian information Criterion or leave-one-out cross validation. In the following analysis we set 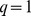, as in other fMRI studies [18]. From now on we refer to this data-driven method as PCGC*^d^*.

The statistical significance of Granger causality value was estimated under the null hypothesis of zero influence, with a standard F-test on the restricted and unrestricted AR model [39].

In order to cope with extra-large data sets, such as voxel-wise fMRI data, an additional strategy to reduce the number of conditioning variables is in order. In this study it is proposed to make use of the community structure of the data. This procedure, indicated as PCGC*^t^*, exploits a hierarchical partition, at two resolutions, of the brain signal. It consists of the following steps:

[1]. Considering each potential driver voxel 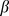, the whole ensemble of voxels (excluding 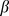) *S* is divided into 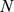 systems: 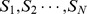, such as the signal for the 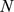 systems is obtained aggregating voxels inside each system 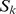 resulting in 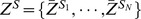.

[2]. Each system is further partitioned into subsystems 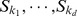, such that now the signal within the subsystems of 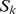 is given by 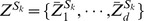, where 
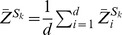
, being 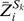 the mean signal of the variables 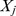 belonging to the subsystem 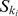.

[3]. If 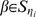, then 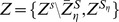, and 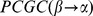 is calculated following Eq.(1).

This strategy is justified by the following assumptions:

Let us consider PCGC*^t^* in the restricted and unrestricted regression models:
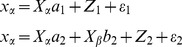
(2)where 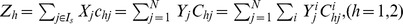; 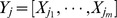, 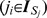; 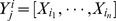, 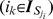; 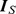, 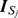 and 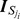 are the index of 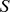, system 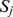 and subsystem 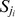 respectively; and 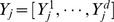.

For voxel-wise analysis, excluding the special case in which 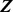 is a small subset containing all the informative variables, the observation is always much smaller than the number of predictors in Eq. 2, resulting in a singular matrix in the computation of the regression coefficients. Moreover, predictors will also face a high degree of multicollinearity (predictors too are redundant). As a consequence estimation of regression coefficients in CGC may change erratically in response to small changes in the data.

According to our algorithm, the coefficients of 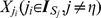 will have the same given weight; different weights will be assigned to the coefficients of 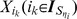, thus
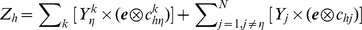
where 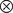 denotes the Kronecker product and 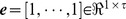, 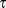 is changed according to the dimension of 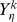 and 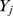.

So, in the proposed algorithm, even if we only consider a few conditioning variables 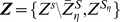, we are potentially taking into account all the information needed to partial out possible indirect causal influences, and avoiding multicollinearity in regression analysis models.

In order to achieve effectiveness and feasibility of the proposed scheme, the predictors should be reasonably aggregated into groups, ensuring that they contribute with approximately equal weights to the dependent variable. Since the construction of a pair-wise correlation matrix will yield indications on the likelihood that predictor variables are multicollinear/redundant, we can group the predictors after detecting community structure from the correlation matrix. We then average the predictors which contain the redundant information about the dependent variable to avoid overfitting in regression analysis model. Considering that spatially connected voxels will most likely display similar BOLD signal, we can find community structure on a coarse resolution under the local mean-field assumption.

### Detection of the Conditioning Dataset

#### Community detection

In order to reduce the dimensionality of the set 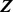 of variables to include in the conditional analysis we explored its community distribution.

First, the preprocessed functional images were parcellated into 90 (45 for each hemisphere) non-cerebellar anatomical regions of interest (ROIs) using automated anatomical labeling (AAL) template [40]. This parcellation scheme is referred to as AAL-90. Considering that the range of nodal scale and the difference in template parcellations may affect the results of community detection [41], we also used a high-resolution parcellation scheme with 512 and 1024 micro ROIs [42], [43]. Specifically, we generated smaller ROIs of approximately identical size across both hemispheres by subdividing each region of the low-resolution AAL-90 template into a set of sub-regions. These parcellation schemes are referred to as AAL-512 and AAL-1024. The study-specific functional volume mask was superposed to the AAL-90/512/1024 templates.

Then, the time series from each ROI *i* and *j* were used to calculate the pairwise Pearson correlation matrix *R* = (*r_ij_*) for each subject. This matrix was averaged across all subjects and its community structure was explored. As negative weights play a controversial role in network organization [44], for this study the absolute values of the averaged matrix were considered. The Louvain algorithm for modularity detection was run 10^4^ times, and the solution producing the highest *Q* was selected as the representative modular partition, where modularity *Q* was defined as [45]:
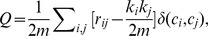



Where 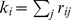, 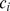
is the community to which vertex *i* is assigned, 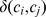 is the Kronecker delta, and 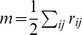. According to the PCGC*^t^* algorithm, these large modules were further divided into smaller sub-modules according to the strategy described above.

#### Statistical analysis of Z

**Figure 1:**
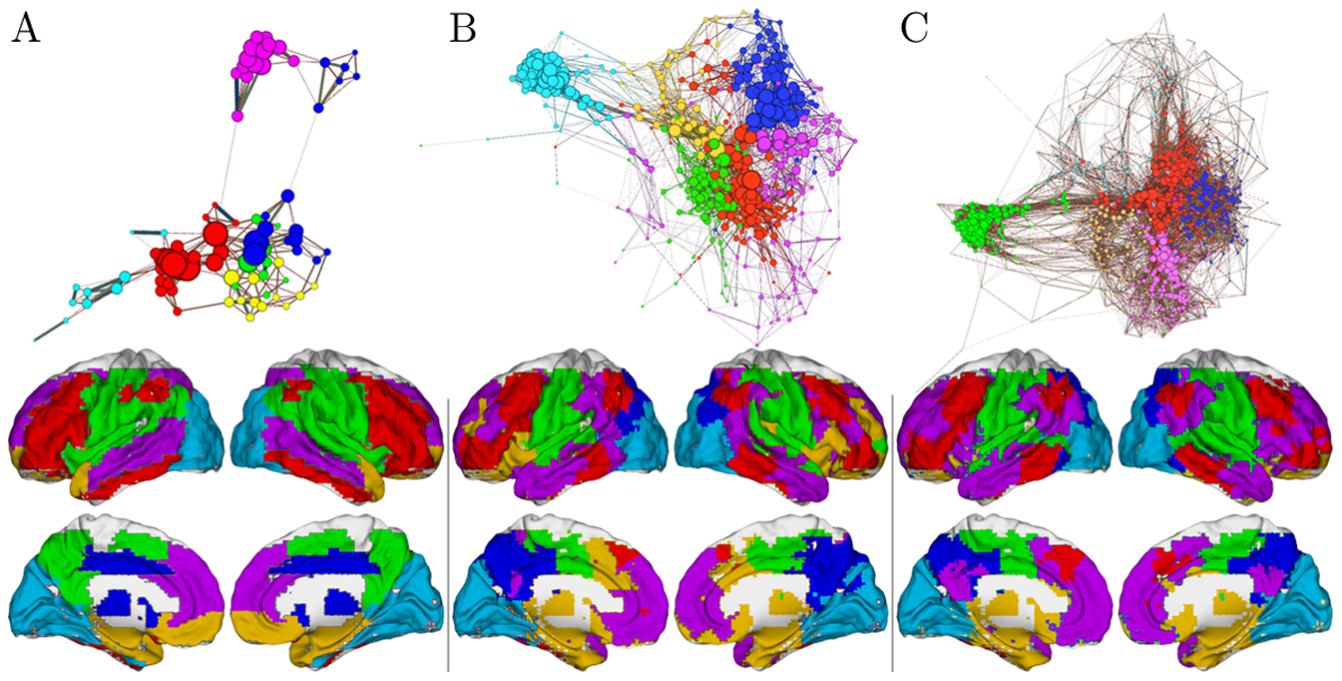
The functional connectome: layout and communities. The full brain contains about 43413 3-mm cubic voxels for AAL-90 (A), AAL-512(B) and AAL-0124 (C) template. On average 6 functional communities were found. They are colored distinctly within multiple axial slices and 3D rendered on MNI152 standard brain surface (Bottom). To highlight the overall layout at a sparse 2% connection density, the functional connectome was further visualized as a network layout with the same colors (Top).

**Figure 2:**
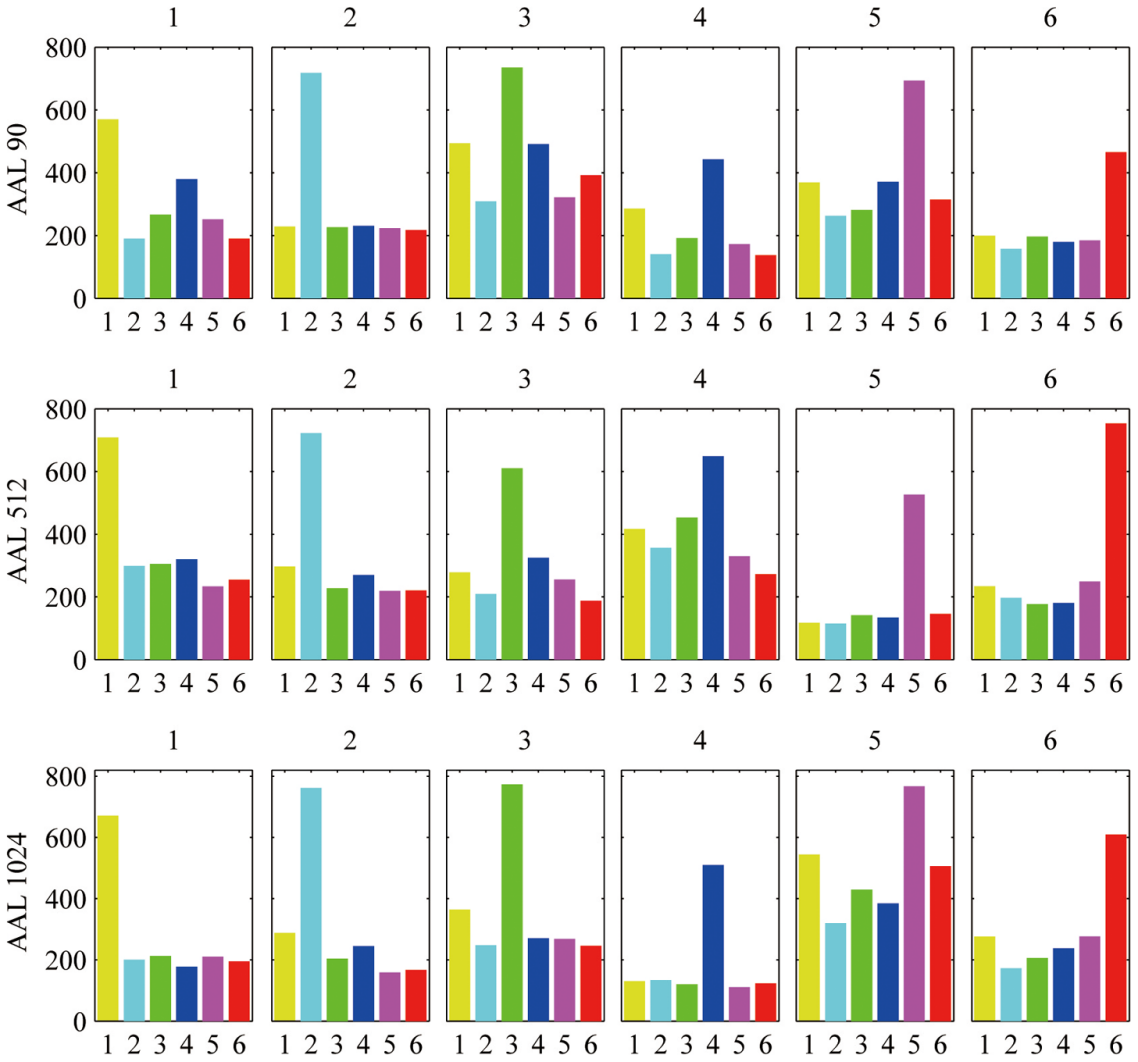
Distributions of the most informative variables contained in the set 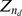 across communities.

Following the identification of modules from the mean correlation matrix (see Fig. 1), further analysis was performed on the distributions of ***Z*** according to modular structure. The distributions of the first *n_d_* variables (obtained from greedy algorithm) in the partitioned module are reported in Fig. 2 in which it’s evident that the most informative variables for each candidate driver come mainly from the same partition, but also, with no major differences in proportion, from the other modules.

#### Effect of including the driver variable in Z

The formulation of PCGC*^t^* requires that the driver variable 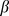 is excluded before partitioning the system. While this step is absolutely necessary at large scale, when working with time series from individual voxels one can suppose that the results will not be dramatically affected since its effect will be most likely averaged out. Including the driver variable is computationally very advantageous, saving time in the partition step.

To validate this hypothesis, we propose a test to evaluate how the presence of the candidate driver variable affects the result of the voxel-wise PCGC*^t^* analysis. Firstly, the correlation between the average signal 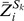 of subsystem 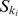 and its individual voxels is computed (see the distribution of these values in Fig. S1). Then, for every subsystem, a driver voxel 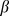 yielding the maximum value of 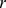 is chosen, and PCGC*^t^* is computed including it in the subsystem ***Z***. This modified approach is called PCGC*^ti^*.

### Seed-based Granger Causality

**Figure 3:**
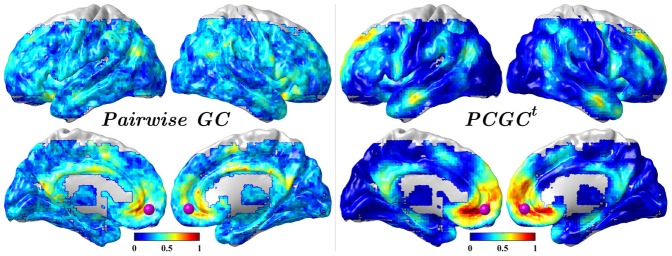
Reproducibility of the causal flow from mPFC (purple sphere) when using pairwise GC(left) and PCGC*^t^* (right) under 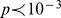. Relative frequency with which a voxel was selected as a hub for outgoing information.

As a representative example, medial prefrontal cortex (mPFC, MNI coordinates [0, 52, −6] with sphere 6mm diameter, see Fig. 3) was used as the seed ROI. This choice is motivated by the evidence of it being a hub sending out information in default mode network [46]. The set ***Z*** of conditioning variables was chosen on AAL-1024 template. Causal interaction was investigated by mapping the influence from the source to voxels in the rest of the brain. Indirect influences will be misleadingly considered as direct in the traditional pairwise GC analysis, which was computed for comparison and validation.

### Voxel-wise Granger Causality

**Figure 4:**
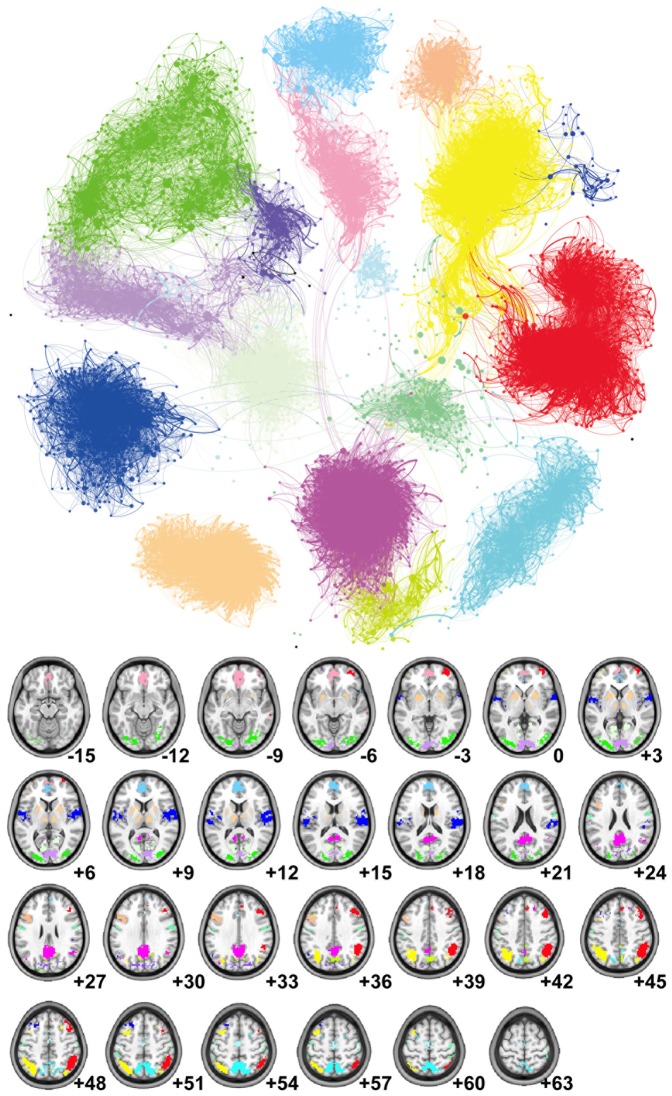
Visualization of the group-level voxel-wise directed graph. Upper panel: layout at a sparse 2.4% connection density, 8156 voxels with degree>11 are displayed; the 17 bigger communities (detected from the directed network at group level) are indicated by different colors. Lower panel: the spatial distributions of the voxels in the upper panel are mapped on the anatomical image with the same colors.

To construct the voxel-wise Granger causality network, the PCGC conditioning variables ***Z*** were individuated using the AAL-1024 based community structure. Specifically, the time series for each voxel were extracted from the HRF-deconvolved rs-fMRI data to calculate a PCGC matrix 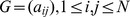 (N is the number of voxels), where 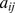 is the GC value between the *i*- and *j*-th voxels. A visualization of group level voxel-wise directed graph resconstructed by PCGC is reported in Fig. 4. Considering that the graph G is directed, all topological properties were calculated on both incoming and outgoing matrix. Graph theoretical analyses were carried out on the EC network using the MatlabBGL package (https://code.launchpad.net/matlab-bgl).

### Centrality Indices

Degree centrality (DC) is the sum of the weights of edges connected to a node, i.e. 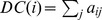. Nodes with high DC can be considered as hubs for information integration.

Betweenness centrality (BC) is a measure based on shortest paths, widely used in complex network analysis. Nodes with high BC are important in managing the flow of information in the graph due to the fact that they have a high probability to occur on a randomly chosen shortest path between two randomly chosen nodes.

Clustering coefficient (CC) is defined as the number of connections among the neighbors of a particular node. It reflects the local efficiency of information transfer in the graph. A high CC along with a small characteristic path length indicates “small-world” architecture, reflecting regional hubs with long-distance connections and high clustering within each of them.

#### Normalized nodal parameters

We calculated the normalized nodal parameters as in the following formula [47]:
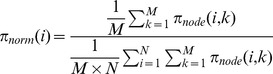
where 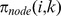 is an integrated nodal parameter (BC, CC and DC) of node *i* in the network of subject *k*, *M* is the number of networks included in the analysis(*M* = 197) and *N* is the number of nodes.

#### Identification of hubs

The hubs for each node in the brain network were identified according to the following criteria: (1) Node i is a BC-hub if BC_norm_ (i) >mean+SD. (2) Node i is a CC-hub if CC_norm_(i) >mean+SD. (3) Node i is a DC-hub if DC_norm_(i) >mean+SD. To each node was assigned a score between 0 and 3, determined by the total number of hub criteria fulfilled. Voxels showing a hub-score of 2 or 3 (i.e. which were designed hubs for at least two measures) were marked as hub nodes.

### Validations: Simulated Data

The reliability of PCGC*^t^* was validated using simulated data. A benchmark dataset was created based on the following AR(1) model:
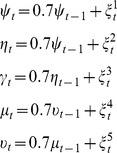
where 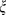 are i.i.d. unit variance Gaussian variables. By construction, 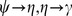 and 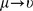. A system of 6*k* time series, where *k = *10 or 20 was constructed as follows. For 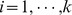:
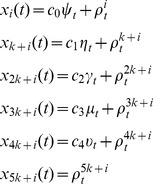
(3)where 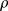 and c are i.i.d. Gaussian variables, 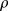 are zero mean and unit variance, c is generated from a Gaussian distribution with mean 0.3 and variance 0.3. Note that the first k variables share the same information corresponding to 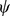 (Module 1), whilst the second k variables share the information corresponding to 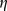 (Module 2). The variables 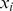 (Module 3), with 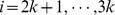, form a group of variables with correlations at equal times, similarly to the group of variables with 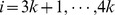 (Module 4) and 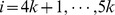 (Module 5). The variables 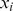, with 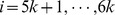 (Module 6), correspond to pure noise. We generated a data set of 5000 time points (in order to get robust statistical significance in the next analysis). Then we evaluated the element-wise GC/PCGC for all pairs of maps. We repeated the simulation 100 times with random values of 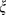, 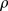 and 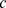 to generate a null distribution; Wilcoxon signed rank test was employed to assess the statistical significance of the links in the repeated simulation result, corrected by family-wise error rate with 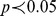.

## Results

Seed-based and voxel-wise Granger causality were evaluated. In the latter case, conditioning variables were obtained after partitioning the data in high-resolution functional connectivity communities. We further report the centrality analyses based on binary directed influence network at voxel-level.

The conditional variables ***Z*** were detected in functional connectomes of different spatial scale, constructed using AAL-90, AAL-512 and AAL-1024 templates. On average 6 communities were detected in each functional connectome (Fig. 1). These results are consistent with previous findings [48], [49]. Further analysis was performed on the distribution of the variables in ***Z*** across the modules. The distribution of 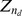 (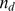 = 10, Fig. 2) according to the partitioned community organization shows that the highest fraction of the predictors in 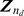 come from the same module of the driver variable, and contributions from other modules are relatively equally distributed.

### Seed-based Granger Causality Mapping

The reproducibility of directed influence from mPFC (seed-to-voxel causality mapping) across all subjects is shown in Fig. 3. The reproducibility is given by the number of subject which showed a significant *F* value, divided by the total number of subjects, for a given voxel. The outgoing information values retrieved with pairwise GC and PCGC*^t^* were relatively consistent (*r* = 0.43). Compared to pairwise GC, the PCGC*^t^* displayed higher reproducibility in medial frontal gyrus, superior frontal gyrus, inferior frontal gyrus, middle temporal gyrus, anterior prefrontal cortex, anterior cingulate, dorsolateral prefrontal cortex, posterior cingulate, precuneus and lower in occipital lobe, cuneus under the same statistical significance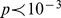.

Moreover, the first 10 most informative voxels for mPFC are shown in Fig. S2, with size proportional to their reproducibility across all subjects. It can be observed that these voxels are distributed not only in proximity of the zone of interest but across the brain, consistently with findings reported for ***Z*** derived when voxel time series were averaged according to AAL-90/512/1024 templates [50].

Concerning the effect of including the driver voxel in ***Z***, we found that PCGC*^ti^* is highly correlated with PCGC*^t^* (minimum correlation 0.993 across all subsystems and subjects, Fig. S3), especially for the statistical significant values, thus indicating that this approximate step has a negligible influence on the accuracy of the method.

### Voxel-wise Granger Causality Network

In Fig. 4 the voxel-wise PCGC*^t^* network is represented using a network layout at 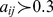 for each subject. This network of directed information is divided in modules which are then mapped on the brain. For a better visualization, only nodes with degree >11 are reported in the figure. The purple cluster, containing the posterior regions of the default mode network is intensely interconnected to other modules. In particular it appears to send directed information to the anterior regions (pink cluster) rather than receiving, providing additional details to previous results on the directionality of information flow in the default mode network [46]. The salmon cluster, containing the thalamus and the putamen, does not have strong connections to the other modules. These results are consistent with those reported in [51], in which it was shown that all midline cortical rich-club nodes (i.e., bilateral precuneus, superior frontal, superior parietal) are connector hubs, playing an important role in between-module connectivity, while subcortical rich-club regions (bilateral thalamus, putamen) play an important role in module structure.

Considering that the graph we focused on is directed, each node’s incoming degree and outgoing degree must also be considered separately [24]. Incoming degree and outgoing degree represent the total number of connections incoming to a node and outgoing from the same node, respectively [52].

**Figure 5:**
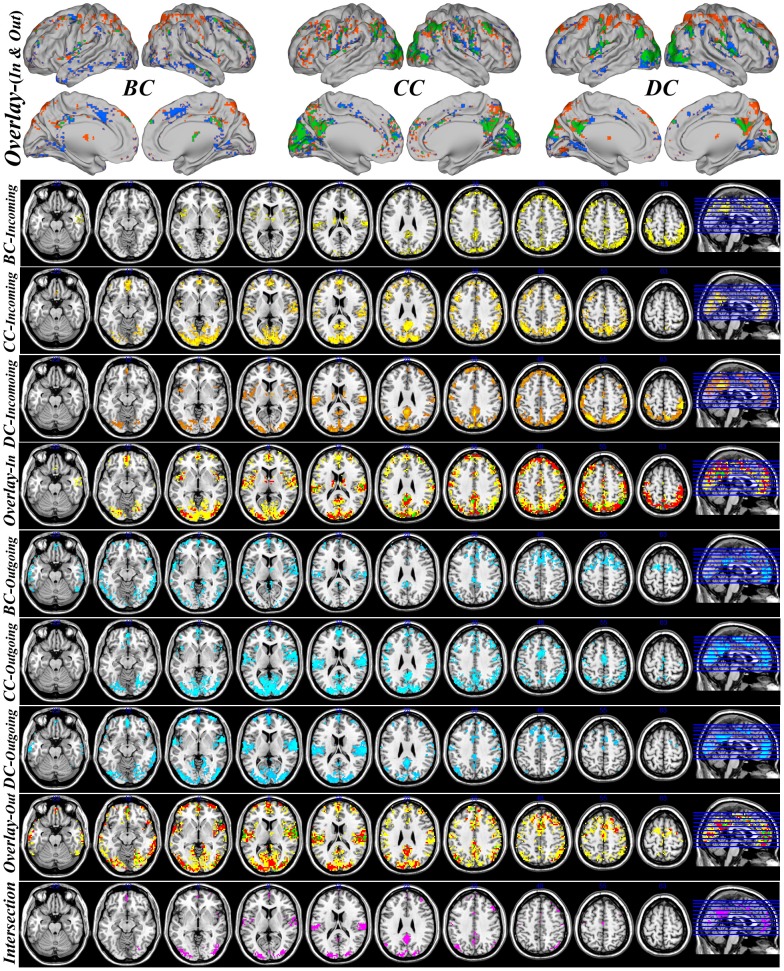
The spatial distribution of hub voxels in a binary graph obtained keeping all the weights higher than a threshold of 0.3, with unitary value, and setting the rest to zero. In the top sagittal views, red indicates the incoming network hubs, blue the outgoing network hubs, while green the common hubs of incoming and outgoing network. Concerning the axial views, 1-3^rd^ (5–7^th^) rows indicate the BC/CC/DC incoming network hubs. In 4^th^ (8^th^) row, yellow indicates incoming (outgoing) regions which are hubs for one measure (hub-score of 1), red indicates incoming (outgoing) regions which are hubs for two measures (hub-score of 2), while green indicates regions which are hubs for all three measures (hub-score of 3). The last row indicates the regions that are at the same time hubs for incoming and outgoing network with hub score of at least 2.

Here only binary graph results with fixed threshold 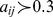 and a minimum cluster size of 27 contiguous voxels were reported. The spatial distributions of the weighted graphs are similar (Fig. S4). Based on normalized nodal parameters, some consistent regions are identified as hubs (voxel hub-score of 2 or 3) at the same time in the incoming and outgoing directed influence network (Fig. 5): middle occipital gyrus, cuneus, postcentral gyrus, precuneus, associative/secondary visual cortex, cingulate gyrus, superior temporal gyrus, dorsal posterior cingulate cortex, inferior parietal lobule, supramarginal gyrus, transverse temporal gyrus, angular gyrus, primary auditory cortex, middle frontal gyrus, posterior cingulate, precentral gyrus, subcentral area. Most of these regions are involved in the following resting state networks: default mode network (DMN), visual network (VN), auditory network (AN). These results are in line with previous reports studying brain anatomical, functional connectivity networks [15], [53].

Cuneus, precuneus, somatosensory associative cortex, associative visual cortex, superior parietal lobule, cingulate gyrus, inferior parietal lobule, dorsal posterior cingulate cortex were evidenced as hubs for incoming information.

Some regions were consistently identified as hubs of outgoing directed influence: superior temporal gyrus, postcentral gyrus, middle occipital gyrus, transverse temporal gyrus, precentral gyrus, and primary auditory cortex.

### GCD vs. FCD

**Figure 6:**
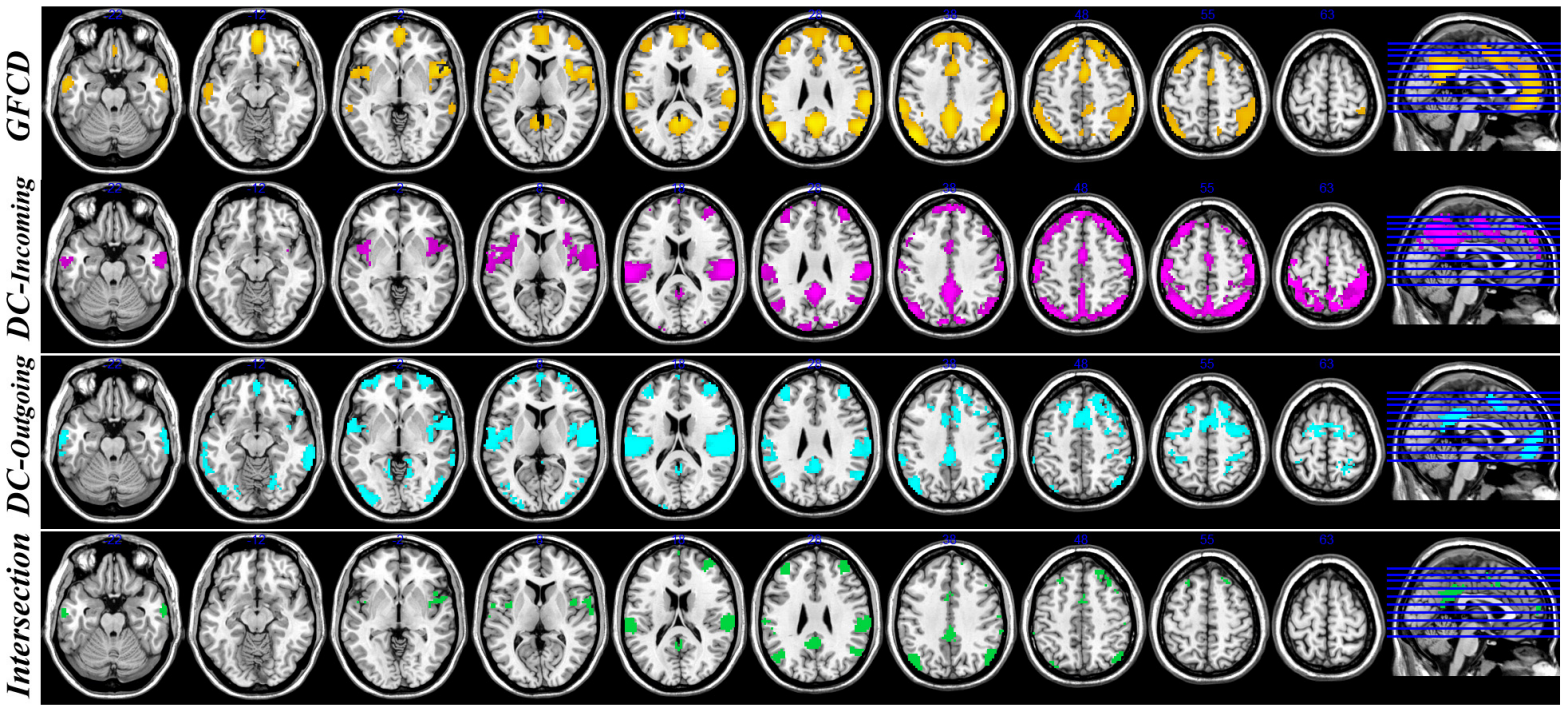
The spatial distribution of hub voxels in a graph binarized with a threshold 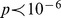. The first row illustrates the spatial distribution of global functional connectivity density hubs. The second (third) row indicates the DC incoming (outgoing) network hubs. The last row indicates the regions which are DC hubs both for FC and EC (incoming/outgoing) networks.

In addition, we compared DC in voxel-wise Granger causality network versus voxel-wise functional connectivity network. For voxel-wise functional connectome, DC was referred to as global FC density (FCD) in previous studies [13]. The FCD map (binary graph at fixed significant threshold 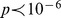) is consistent with previous functional connectivity studies [15], [54]. The incoming and outgoing GCD maps (binary graph results with fixed significant threshold 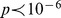) are shown in Fig. 6. The regions showing high DC both for EC (Incoming/Outgoing) and FC are located in middle frontal gyrus, superior frontal gyrus, dorsal frontal cortex, superior temporal gyrus, angular gyrus, supramarginal gyrus, dorsal posterior cingulate cortex, anterior prefrontal cortex, primary auditory cortex, precuneus, insula, posterior cingulate cortex; most of them are part of the DMN system.

### Simulated Validations

**Figure 7:**
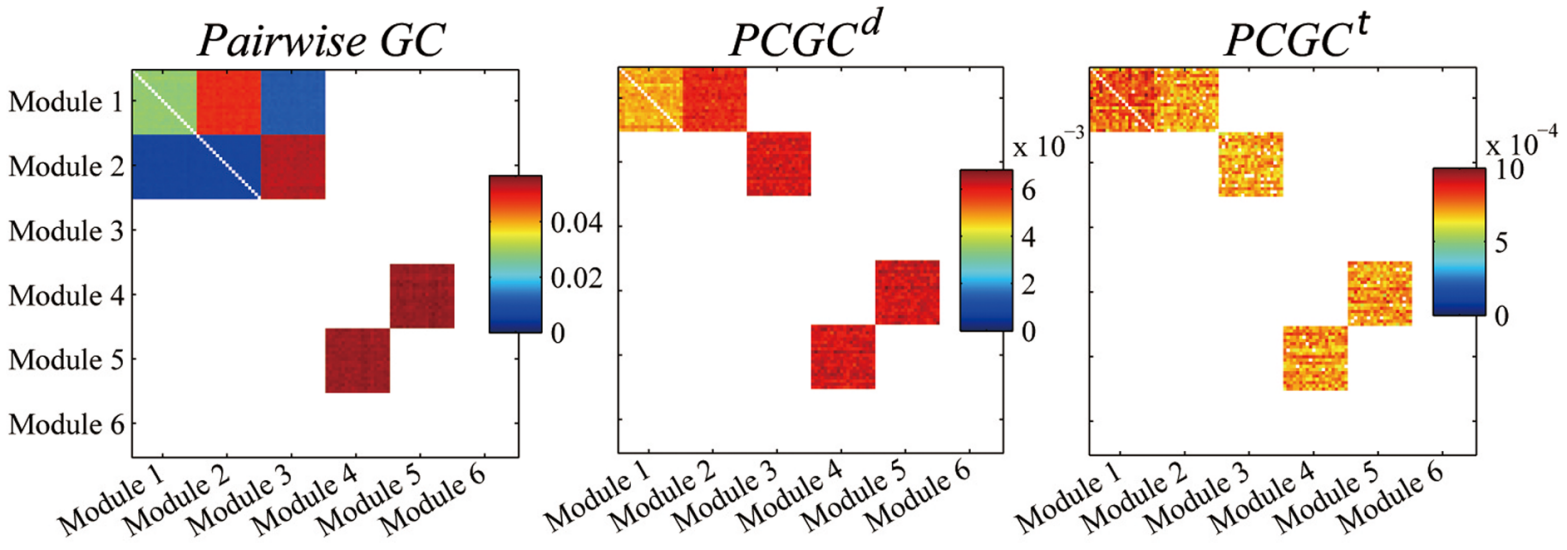
Results of the different PCGC algorithms on simulated data (k = 20). Left: pairwise GC, center: PCGC*^d^*, right: PCGC*^t^*.

We simulated data according to Eq.2 with *k* = 10, 20. The resulting modules when *k* = 20 are reported in Fig. 7 (similar results are obtained with *k* = 10). Pairwise GC and PCGC analysis were performed with model order equal to 1. PCGC*^t^* and PCGC*^d^* all successfully revealed the ground truth in both cases, while pairwise GC detected false positives from Module 2 to Module 1, and from Module 1 to Module 3. The *n_d_* = 10 for PCGC*^d^* analysis is determined by the knee of the curve of the information gain when an additional variable is used for conditioning (Fig. S5 right).

## Discussion

Large-scale integration of information across brain regions is investigated by both functional and anatomical connectomes. In this study, to extend human brain connectomic repertoire, we first constructed the effective connectivity network using voxel-wise Granger causality on resting-state fMRI data. To cope with dimensionality issues for voxel-wise Granger causality and to decouple the neuronal activity and hemodynamic responses of resting-state fMRI, we proposed the partially conditioned Granger causality (PCGC) and blind deconvolution using the spontaneous events detected in BOLD signal. The convergence and divergence of hub regions between functional and effective connectivity network were documented.

### Directed Network Centrality Mapping

Specific network centrality measures have been primarily focused on the identification of the human brain hubs at regional [9], [55] and voxel level [13], [15], [54], [56]–[58]. Brain hubs take a central position in a network and play a crucial role in fast transfer and efficient integration of information across the human connectome [3]. In this study, hubs of directed brain network were generally identified by high levels of degree centrality, betweenness centrality, and clustering coefficient [3], [58]. As an addition to previous findings in structural and functional connectomes, here for the first time the voxel-wise centrality-based characteristics of information flow in the human brain directed network was reported. Some regions have been found to be consistently hubs across various modalities (e.g., fMRI vs. DTI) and different dynamical connectivity approaches (FC vs. EC), such as posterior cingulate cortex, precuneus, medial prefrontal cortex, lateral parietal and temporal cortex, insula. Also, some regions displayed remarkable differences (e.g., cuneus), due to the specific measure of centrality [15], the parcellation scale [41] and brain connectivity definition [53] employed. Nonetheless, our findings suggest that higher order cortical association regions acted as pivotal incoming or outgoing hubs, maintaining information flow even in resting state.

Although pivotal hubs have already been found within single resting-state network [46], among multiple networks [5], [19], and even in large-scale whole brain network [4], [24], uncovering voxel-wise centrality hubs on directed networks is particularly challenging. Efficient algorithms to estimate voxel-wise centralities are still under development [59], while computation of the intermediate directed connectivity matrix (∼10^9^ elements) involves accuracy and efficiency problems. In the present work, we proposed a novel approach, PCGC*^t^*, to remove indirect interactions in large multivariate datasets.

### Partial Conditioning Technique

It has been recently proposed [25] that partial conditioning on a small number of the most informative variables for the driver node is sufficient to obtain a reliable estimate of the directed connectivity, especially when the pattern of causalities is sparse. This approach not only allows a much faster calculation of Granger causality matrix, but also a more accurate one, where a fully multivariate approach would incur in curse of dimensionality and in underestimation of influences due to the presence of redundancy. Anatomical studies have shown that axonal connectivity of the cortex is generally sparse [53], functional connectivity studies have been shown that the human brain is a highly clustered and redundancy complex system. Furthermore, the information gain plots reflect that the most informative variables for driver node were confined to small number of nodes or components. These evidences provide the idea to construct voxel-wise EC network by uses of partial conditioning technique.

In a recent study we have shown that the relative information gain (and thus the number of variables to condition on) is not affected by the time between successive scans (TR) [26], even though data with shorter TR contain more absolute information. Here we further examined how template size affects the information gain [60]. However, with lower scale template (such as AAL 512 and AAL 1024, and in general when the number of variables is larger than the number of samples), the residual redundancy will prevent a further decrease of the information gain after a local minimum. On the other hand, when ***Z*** is built from the aggregated signal according to community structure, this phenomenon disappears (Fig. S5).

The statistical analysis of 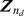
provides the evidence that the most informative variables for the candidate driver mostly come from the community to which it belongs and are uniformly distributed within the rest of communities. This may give an additional explanation for the number of variables for which the curve of the information gain shows a knee, corresponding to the case in which relevant information is picked across all the communities. The joint information collected from the information gain curve, and the sensitivity and specificity of the greedy searching approach, one can choose the most convenient number of variables to include in the conditioning dataset. In the present study we set *n_d = _*10.

PCGC*^d^* method is similar to LASSO based full-brain AR model [61]–[63], only including a few variables to predict the other ones. Compared to PCGC*^d^*, PCGC*^t^* uses all the information from the conditional variables, and a proportional distribution of weight values for conditional variable in AR model are fixed a priori according to the community parcellation results.

### Methodological Considerations and Limitations

On average 6.7 min/subject were required to complete a network, running on Windows 7 (64 bit), Processor: Intel(R) Core(TM) i5-2400 CPU @ 3.10GHz, Installed memory (RAM): 16.0 GB.

In the simulated model, we did not consider the effect of time series length. We only chose a fixed value of the data length which ensured a robust significant causal inference. In addition, the simulated is not meant to reproduce complex brain activity, it is rather a controlled benchmark to be used for a proof of concept.

Community structure revealed by grouping the first 10 most informative contribution regions across all subjects at large scale parcellation (AAL-90) shows that there is a well distributed spatial organization of the set of conditioning variables ***Z*** [50]. Based on the above evidence, the distribution the variables in ***Z*** was further explored in the current study, and community organization derived from correlation matrix was reported as stable across three parcellations with increasing spatial resolution (AAL-90, AAL-512, AAL-1024), but it still remains to be validated how the performance of PCGC*^t^* is affected by inter- and intra-subject variability of the community structure [64].

It is also worth to note that apart from directed connectivity, the problem of conditional dependencies affects as well correlation-based undirected (functional) connectivity, and a generalization of the approach proposed here to the latter case could be in order, and straightforward.

Here we reported the findings based on binary network, such as FCD. However, given that weighted networks contain information about connection strength that reflects heterogeneity in capacity and intensity of connections, these latter could be more indicated for brain connectome representation. For a cross-validation of our results, we additionally used Granger causality strength to identify brain hubs based on weighted effective connectivity network (see Figs. S4, S6 and S7). These results are in accordance with the ones described in the main text.

Finally, for cross-validation of threshold selection, we used additional thresholds to evaluate the stability of the hubs organization in the effective networks (see Figs. S6 and S7), obtaining a general consistence across all the values.

To summarize, we proposed a an approach to perform partially conditioned Granger causality rooted in information theory and graph-theory analysis, coupled to a blind deconvolution technique based on point process analysis to reconstruct the voxel-wise effective connectome of the human brain. We put in evidence for the first time the voxel-wise hubs of incoming and outgoing information, as a complement to previous results on functional and anatomical connectomes. Analogies and differences with these networks have been presented and discussed. Our findings could open the way to a new description of global organization and information influence of brain function in terms of the Granger causality density.

## Supporting Information

Figure S1 **Distribution of Pearson correlation r between each voxel and the mean signal of its community (according to the community structure retrieved from AAL-1024).**
(TIF)

Figure S2 **Spatial distribution of the** ***n_d_*** **= 10 most informative voxels for seed region mPFC (MNI coordinate: [0 52 −6], 6mm- diameter sphere, blue).** The size and color of the sphere denote the relative frequency with which a given voxel was selected.
(TIF)

Figure S3 **Log-log plot of PCGC** ***^ti^*** **and PCGC** ***^t^*** **. Inset, linear plot.**
(TIF)

Figure S4 **The spatial distribution of hub voxels of the weighted graph obtained keeping all the weights higher than a threshold of 0.3, with their value, and setting the rest to zero.** In the top sagittal views, red indicates the incoming network hubs, blue the outgoing network hubs, while green the common hubs of incoming and outgoing network. Concerning the axial views, 1–3^rd^ (5–7^th^) rows indicate the BC/CC/DC incoming network hubs. In 4^th^ (8^th^) row, yellow indicates incoming (outgoing) regions which are hubs for one measure (hub-score of 1), red indicates incoming (outgoing) regions which are hubs for two measures (hub-score of 2), while green indicates regions which are hubs for all three measures (hub-score of 3). The last row indicates the regions that are at the same time hubs for incoming and outgoing network with hub score of at least 2.
(TIF)

Figure S5
**The mutual information gain (Δ** ***y*** **), when the (** ***n_d_*** **+1)-th variable is included, is plotted versus** ***n_d_*** **.** The information gain is averaged over all the variables. Left: the conditioning set 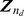 is calculated from the raw signal extracted from AAL-90/512/1024 template; Top right: 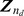 is calculated on the signal extracted from each community; Right: curves for the simulated dataset;(TIF)

Figure S6
**CC hubs distribution under different thresholds (rows from top to down,** 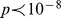 **, 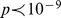, 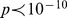, 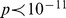, 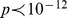,** ***a_ij_*** **>0.3 ).** Top left, Incoming network (binary graph) CC hubs; Top right, Incoming network (weighted graph) CC hubs; Bottom left, Outgoing network (binary graph) CC hubs; Bottom right, Outgoing network (weighted graph) CC hubs.(TIF)

Figure S7
**DC hubs distribution under different thresholds (rows from top to down,** 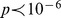 **, 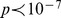,** 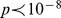 **, 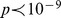, 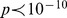, 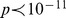, 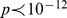,** ***a_ij_*** **>0.3 ).** Top left, Incoming network (binary graph) DC hubs; Top right, Incoming network (weighted graph) DC hubs; Bottom left, Outgoing network (binary graph) DC hubs; Bottom right, Outgoing network (weighted graph) DC hubs.(TIF)

## References

[1] Sporns O (2013) Network attributes for segregation and integration in the human brain. Curr Opin Neurobiol 23: 162–171. 23294553 10.1016/j.conb.2012.11.015

[2] Friston KJ (2011) Functional and effective connectivity: a review. Brain Connect 1: 13–36. 22432952 10.1089/brain.2011.0008

[3] Bullmore E, Sporns O (2009) Complex brain networks: graph theoretical analysis of structural and functional systems. Nat Rev Neurosci 10: 186–198. 19190637 10.1038/nrn2575

[4] Yan C, He Y (2011) Driving and driven architectures of directed small-world human brain functional networks. PLoS One 6: e23460. 21858129 10.1371/journal.pone.0023460 PMC3155571

[5] Liao W, Mantini D, Zhang Z, Pan Z, Ding J, et al (2010) Evaluating the effective connectivity of resting state networks using conditional Granger causality. Biol Cybern 102: 57–69. 19937337 10.1007/s00422-009-0350-5

[6] Liu YY, Slotine JJ, Barabasi AL (2011) Controllability of complex networks. Nature 473: 167–173. 21562557 10.1038/nature10011

[7] Richiardi J, Eryilmaz H, Schwartz S, Vuilleumier P, Van De Ville D (2011) Decoding brain states from fMRI connectivity graphs. Neuroimage 56: 616–626. 20541019 10.1016/j.neuroimage.2010.05.081

[8] Eguiluz VM, Chialvo DR, Cecchi GA, Baliki M, Apkarian AV (2005) Scale-free brain functional networks. Phys Rev Lett 94: 018102. 15698136 10.1103/PhysRevLett.94.018102

[9] Salvador R, Suckling J, Coleman MR, Pickard JD, Menon D, et al (2005) Neurophysiological architecture of functional magnetic resonance images of human brain. Cereb Cortex 15: 1332–1342. 15635061 10.1093/cercor/bhi016

[10] Buckner RL, Sepulcre J, Talukdar T, Krienen FM, Liu H, et al (2009) Cortical hubs revealed by intrinsic functional connectivity: mapping, assessment of stability, and relation to Alzheimer’s disease. J Neurosci 29: 1860–1873. 19211893 10.1523/JNEUROSCI.5062-08.2009 PMC2750039

[11] Cole MW, Pathak S, Schneider W (2010) Identifying the brain’s most globally connected regions. Neuroimage 49: 3132–3148. 19909818 10.1016/j.neuroimage.2009.11.001

[12] Hayasaka S, Laurienti PJ (2010) Comparison of characteristics between region-and voxel-based network analyses in resting-state fMRI data. Neuroimage 50: 499–508. 20026219 10.1016/j.neuroimage.2009.12.051 PMC2824075

[13] Tomasi D, Volkow ND (2010) Functional connectivity density mapping. Proceedings of the National Academy of Sciences 107: 9885–9890. 10.1073/pnas.1001414107 PMC2906909 20457896

[14] Power JD, Cohen AL, Nelson SM, Wig GS, Barnes KA, et al (2011) Functional network organization of the human brain. Neuron 72: 665–678. 22099467 10.1016/j.neuron.2011.09.006 PMC3222858

[15] Zuo XN, Ehmke R, Mennes M, Imperati D, Castellanos FX, et al (2012) Network centrality in the human functional connectome. Cereb Cortex 22: 1862–1875. 21968567 10.1093/cercor/bhr269

[16] Friston K, Moran R, Seth AK (2013) Analysing connectivity with Granger causality and dynamic causal modelling. Curr Opin Neurobiol 23: 172–178. 23265964 10.1016/j.conb.2012.11.010 PMC3925802

[17] Granger CW (1969) Investigating causal relations by econometric models and cross-spectral methods. Econometrica: Journal of the Econometric Society: 424–438.

[18] Roebroeck A, Formisano E, Goebel R (2005) Mapping directed influence over the brain using Granger causality and fMRI. Neuroimage 25: 230–242. 15734358 10.1016/j.neuroimage.2004.11.017

[19] Sridharan D, Levitin DJ, Menon V (2008) A critical role for the right fronto-insular cortex in switching between central-executive and default-mode networks. Proceedings of the National Academy of Sciences 105: 12569–12574. 10.1073/pnas.0800005105 PMC2527952 18723676

[20] Uddin LQ, Kelly AM, Biswal BB, Castellanos FX, Milham MP (2009) Functional connectivity of default mode network components: correlation, anticorrelation, and causality. Hum Brain Mapp 30: 625–637. 18219617 10.1002/hbm.20531 PMC3654104

[21] Friston K (2011) Dynamic causal modeling and Granger causality Comments on: the identification of interacting networks in the brain using fMRI: model selection, causality and deconvolution. Neuroimage 58: 303–305; author reply 310–301. 10.1016/j.neuroimage.2009.09.031 PMC3183826 19770049

[22] Roebroeck A, Formisano E, Goebel R (2011) The identification of interacting networks in the brain using fMRI: Model selection, causality and deconvolution. Neuroimage 58: 296–302. 19786106 10.1016/j.neuroimage.2009.09.036

[23] Zhou Z, Chen Y, Ding M, Wright P, Lu Z, et al (2009) Analyzing brain networks with PCA and conditional Granger causality. Hum Brain Mapp 30: 2197–2206. 18830956 10.1002/hbm.20661 PMC6871256

[24] Liao W, Ding J, Marinazzo D, Xu Q, Wang Z, et al (2011) Small-world directed networks in the human brain: multivariate Granger causality analysis of resting-state fMRI. Neuroimage 54: 2683–2694. 21073960 10.1016/j.neuroimage.2010.11.007

[25] Marinazzo D, Pellicoro M, Stramaglia S (2012) Causal information approach to partial conditioning in multivariate data sets. Comput Math Methods Med 2012: 303601. 22675400 10.1155/2012/303601 PMC3364562

[26] Wu GR, Liao W, Stramaglia S, Ding JR, Chen H, et al (2013) A blind deconvolution approach to recover effective connectivity brain networks from resting state fMRI data. Med Image Anal 17: 365–374. 23422254 10.1016/j.media.2013.01.003

[27] Power JD, Barnes KA, Snyder AZ, Schlaggar BL, Petersen SE (2012) Spurious but systematic correlations in functional connectivity MRI networks arise from subject motion. Neuroimage 59: 2142–2154. 22019881 10.1016/j.neuroimage.2011.10.018 PMC3254728

[28] Satterthwaite TD, Wolf DH, Loughead J, Ruparel K, Elliott MA, et al (2012) Impact of in-scanner head motion on multiple measures of functional connectivity: relevance for studies of neurodevelopment in youth. Neuroimage 60: 623–632. 22233733 10.1016/j.neuroimage.2011.12.063 PMC3746318

[29] Van Dijk KR, Sabuncu MR, Buckner RL (2012) The influence of head motion on intrinsic functional connectivity MRI. Neuroimage 59: 431–438. 21810475 10.1016/j.neuroimage.2011.07.044 PMC3683830

[30] Carp J (2013) Optimizing the order of operations for movement scrubbing: Comment on Power et al. Neuroimage 76: 436–438. 22227884 10.1016/j.neuroimage.2011.12.061

[31] Handwerker DA, Gonzalez-Castillo J, D’Esposito M, Bandettini PA (2012) The continuing challenge of understanding and modeling hemodynamic variation in fMRI. Neuroimage 62: 1017–1023. 22366081 10.1016/j.neuroimage.2012.02.015 PMC4180210

[32] Valdes-Sosa PA, Roebroeck A, Daunizeau J, Friston K (2011) Effective connectivity: influence, causality and biophysical modeling. Neuroimage 58: 339–361. 21477655 10.1016/j.neuroimage.2011.03.058 PMC3167373

[33] Deco G, Jirsa VK (2012) Ongoing cortical activity at rest: criticality, multistability, and ghost attractors. J Neurosci 32: 3366–3375. 22399758 10.1523/JNEUROSCI.2523-11.2012 PMC6621046

[34] Petridou N, Gaudes CC, Dryden IL, Francis ST, Gowland PA (2012) Periods of rest in fMRI contain individual spontaneous events which are related to slowly fluctuating spontaneous activity. Hum Brain Mapp. 10.1002/hbm.21513 PMC6869909 22331588

[35] Davis B, Jovicich J, Iacovella V, Hasson U (2013) Functional and Developmental Significance of Amplitude Variance Asymmetry in the BOLD Resting-State Signal. Cereb Cortex. 10.1093/cercor/bhs416 23329729

[36] Tagliazucchi E, Balenzuela P, Fraiman D, Chialvo DR (2010) Brain resting state is disrupted in chronic back pain patients. Neuroscience letters 485: 26. 20800649 10.1016/j.neulet.2010.08.053 PMC2954131

[37] Tagliazucchi E, Balenzuela P, Fraiman D, Chialvo DR (2012) Criticality in large-scale brain FMRI dynamics unveiled by a novel point process analysis. Front Physiol 3: 15. 22347863 10.3389/fphys.2012.00015 PMC3274757

[38] Glover GH (1999) Deconvolution of impulse response in event-related BOLD fMRI. Neuroimage 9: 416–429. 10191170 10.1006/nimg.1998.0419

[39] Geweke JF (1984) Measures of conditional linear dependence and feedback between time series. Journal of the American Statistical Association 79: 907–915.

[40] Tzourio-Mazoyer N, Landeau B, Papathanassiou D, Crivello F, Etard O, et al (2002) Automated anatomical labeling of activations in SPM using a macroscopic anatomical parcellation of the MNI MRI single-subject brain. Neuroimage 15: 273–289. 11771995 10.1006/nimg.2001.0978

[41] Wang J, Wang L, Zang Y, Yang H, Tang H, et al (2009) Parcellation-dependent small-world brain functional networks: a resting-state fMRI study. Hum Brain Mapp 30: 1511–1523. 18649353 10.1002/hbm.20623 PMC6870680

[42] Fornito A, Zalesky A, Bullmore ET (2010) Network scaling effects in graph analytic studies of human resting-state FMRI data. Front Syst Neurosci 4: 22. 20592949 10.3389/fnsys.2010.00022 PMC2893703

[43] Zalesky A, Fornito A, Harding IH, Cocchi L, Yucel M, et al (2010) Whole-brain anatomical networks: does the choice of nodes matter? Neuroimage 50: 970–983. 20035887 10.1016/j.neuroimage.2009.12.027

[44] Rubinov M, Sporns O (2011) Weight-conserving characterization of complex functional brain networks. Neuroimage 56: 2068–2079. 21459148 10.1016/j.neuroimage.2011.03.069

[45] Newman ME (2006) Modularity and community structure in networks. Proc Natl Acad Sci U S A 103: 8577–8582. 16723398 10.1073/pnas.0601602103 PMC1482622

[46] Jiao Q, Lu G, Zhang Z, Zhong Y, Wang Z, et al (2011) Granger causal influence predicts BOLD activity levels in the default mode network. Hum Brain Mapp 32: 154–161. 21157880 10.1002/hbm.21065 PMC6870036

[47] Tian L, Wang J, Yan C, He Y (2011) Hemisphere-and gender-related differences in small-world brain networks: a resting-state functional MRI study. Neuroimage 54: 191–202. 20688177 10.1016/j.neuroimage.2010.07.066

[48] Meunier D, Lambiotte R, Fornito A, Ersche KD, Bullmore ET (2009) Hierarchical modularity in human brain functional networks. Front Neuroinform 3: 37. 19949480 10.3389/neuro.11.037.2009 PMC2784301

[49] Sepulcre J, Sabuncu MR, Yeo TB, Liu H, Johnson KA (2012) Stepwise connectivity of the modal cortex reveals the multimodal organization of the human brain. J Neurosci 32: 10649–10661. 22855814 10.1523/JNEUROSCI.0759-12.2012 PMC3483645

[50] Wu G, Liao W, Chen H, Stramaglia S, Marinazzo D (2013) Recovering directed networks in neuroimaging datasets using partially conditioned Granger causality. Brain Connectivity 3(3): 294–301. 23530810 10.1089/brain.2013.0142 PMC3685317

[51] van den Heuvel MP, Sporns O (2011) Rich-club organization of the human connectome. J Neurosci 31: 15775–15786. 22049421 10.1523/JNEUROSCI.3539-11.2011 PMC6623027

[52] De Vico Fallani F, Astolfi L, Cincotti F, Mattia D, Marciani MG, et al (2007) Cortical functional connectivity networks in normal and spinal cord injured patients: Evaluation by graph analysis. Hum Brain Mapp 28: 1334–1346. 17315225 10.1002/hbm.20353 PMC6871447

[53] Hagmann P, Cammoun L, Gigandet X, Meuli R, Honey CJ, et al (2008) Mapping the structural core of human cerebral cortex. PLoS Biol 6: e159. 18597554 10.1371/journal.pbio.0060159 PMC2443193

[54] Tomasi D, Volkow ND (2011) Association between functional connectivity hubs and brain networks. Cerebral cortex 21: 2003–2013. 21282318 10.1093/cercor/bhq268 PMC3165965

[55] He Y, Wang J, Wang L, Chen ZJ, Yan C, et al (2009) Uncovering intrinsic modular organization of spontaneous brain activity in humans. PLoS One 4: e5226. 19381298 10.1371/journal.pone.0005226 PMC2668183

[56] Joyce KE, Laurienti PJ, Burdette JH, Hayasaka S (2010) A new measure of centrality for brain networks. PLoS One 5: e12200. 20808943 10.1371/journal.pone.0012200 PMC2922375

[57] Lohmann G, Margulies DS, Horstmann A, Pleger B, Lepsien J, et al (2010) Eigenvector centrality mapping for analyzing connectivity patterns in fMRI data of the human brain. PLoS One 5: e10232. 20436911 10.1371/journal.pone.0010232 PMC2860504

[58] van den Heuvel MP, Mandl RC, Stam CJ, Kahn RS, Hulshoff Pol HE (2010) Aberrant frontal and temporal complex network structure in schizophrenia: a graph theoretical analysis. J Neurosci 30: 15915–15926. 21106830 10.1523/JNEUROSCI.2874-10.2010 PMC6633761

[59] Wink AM, de Munck JC, van der Werf YD, van den Heuvel OA, Barkhof F (2012) Fast eigenvector centrality mapping of voxel-wise connectivity in functional magnetic resonance imaging: implementation, validation, and interpretation. Brain Connect 2: 265–274. 23016836 10.1089/brain.2012.0087

[60] Wu G, Stramaglia S, Marinazzo D (2012) Decomposition of the Transfer Entropy: Partial Conditioning and Informative Clustering. In: Huang T, Zeng Z, Li C, Leung C, editors. Lecture Notes in Computer Science Volume 7663, Springer, Berlin, Heidelberg: 226–233.

[61] Valdes-Sosa PA, Sanchez-Bornot JM, Lage-Castellanos A, Vega-Hernandez M, Bosch-Bayard J, et al (2005) Estimating brain functional connectivity with sparse multivariate autoregression. Philos Trans R Soc Lond B Biol Sci 360: 969–981. 16087441 10.1098/rstb.2005.1654 PMC1854937

[62] Garg R, Cecchi GA, Rao AR (2011) Full-brain auto-regressive modeling (FARM) using fMRI. Neuroimage 58: 416–441. 21439388 10.1016/j.neuroimage.2011.02.074

[63] Tang W, Bressler SL, Sylvester CM, Shulman GL, Corbetta M (2012) Measuring Granger Causality between Cortical Regions from Voxelwise fMRI BOLD Signals with LASSO. PLoS computational biology 8: e1002513. 22654651 10.1371/journal.pcbi.1002513 PMC3359965

[64] Moussa MN, Steen MR, Laurienti PJ, Hayasaka S (2012) Consistency of network modules in resting-state FMRI connectome data. PLoS One 7: e44428. 22952978 10.1371/journal.pone.0044428 PMC3432126

