# Supplementary figures and images for: Mapping the Voxel-Wise Effective Connectome in Resting State fMRI
Source: PLoS One. 2013 Sep 12;8(9):e73670. doi: 10.1371/journal.pone.0073670 (PMC3771991; doi:10.1371/journal.pone.0073670)

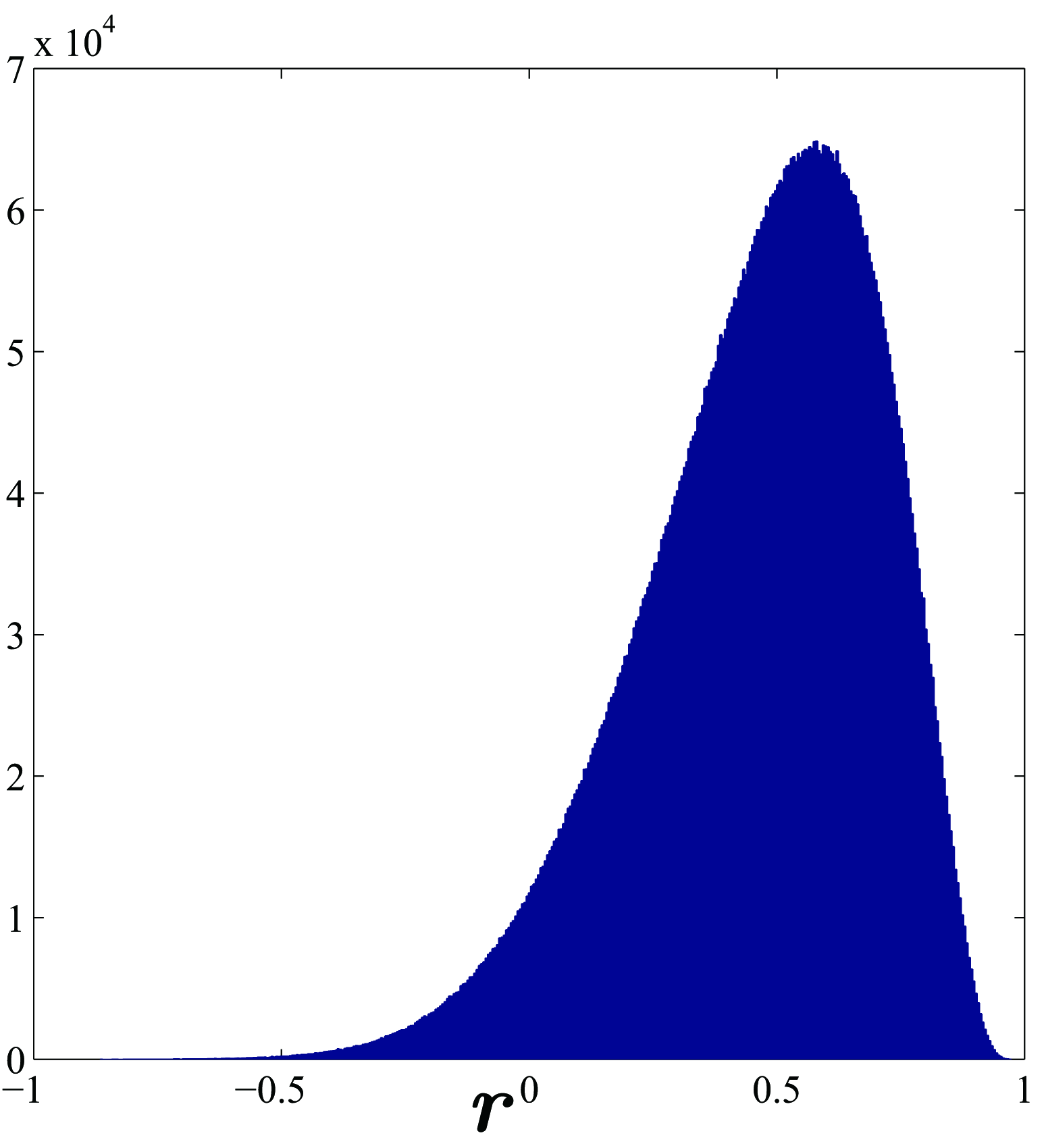

Supplement: Figure S1 — Distribution of Pearson correlation r between each voxel and the mean signal of its community (according to the community structure retrieved from AAL-1024). (TIF) [file pone.0073670.s001.tif]

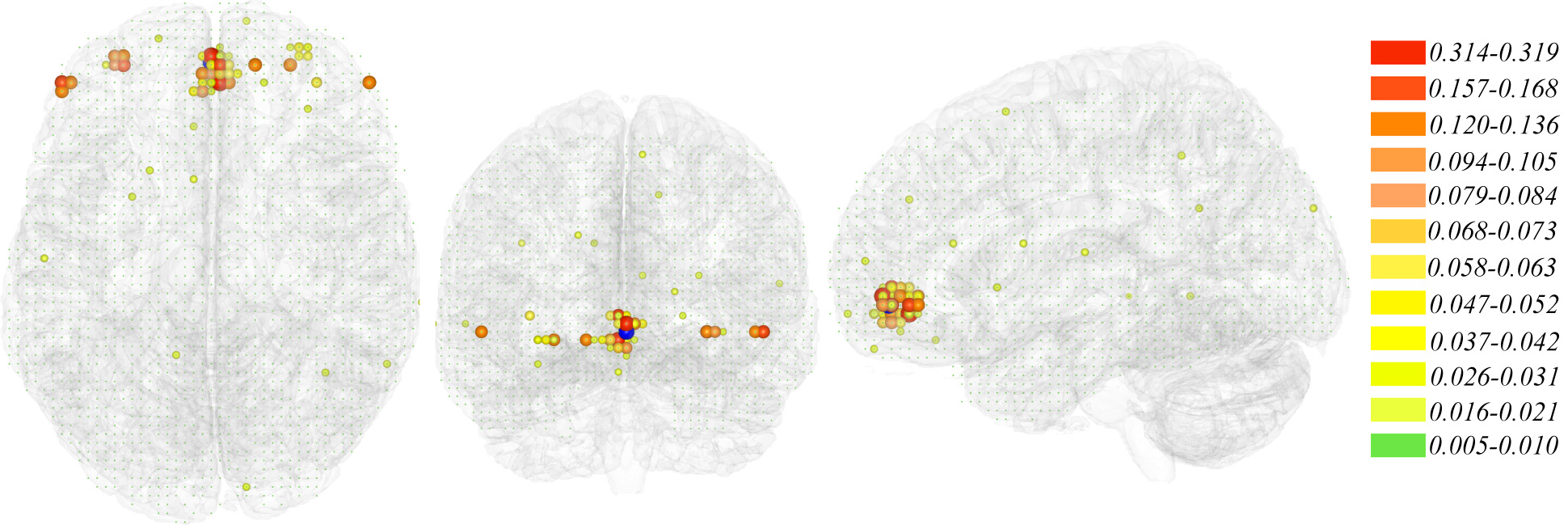

Supplement: Figure S2 — Spatial distribution of the nd = 10 most informative voxels for seed region mPFC (MNI coordinate: [0 52 −6], 6mm- diameter sphere, blue). The size and color of the sphere denote the relative frequency with which a given voxel was selected. (TIF) [file pone.0073670.s002.tif]

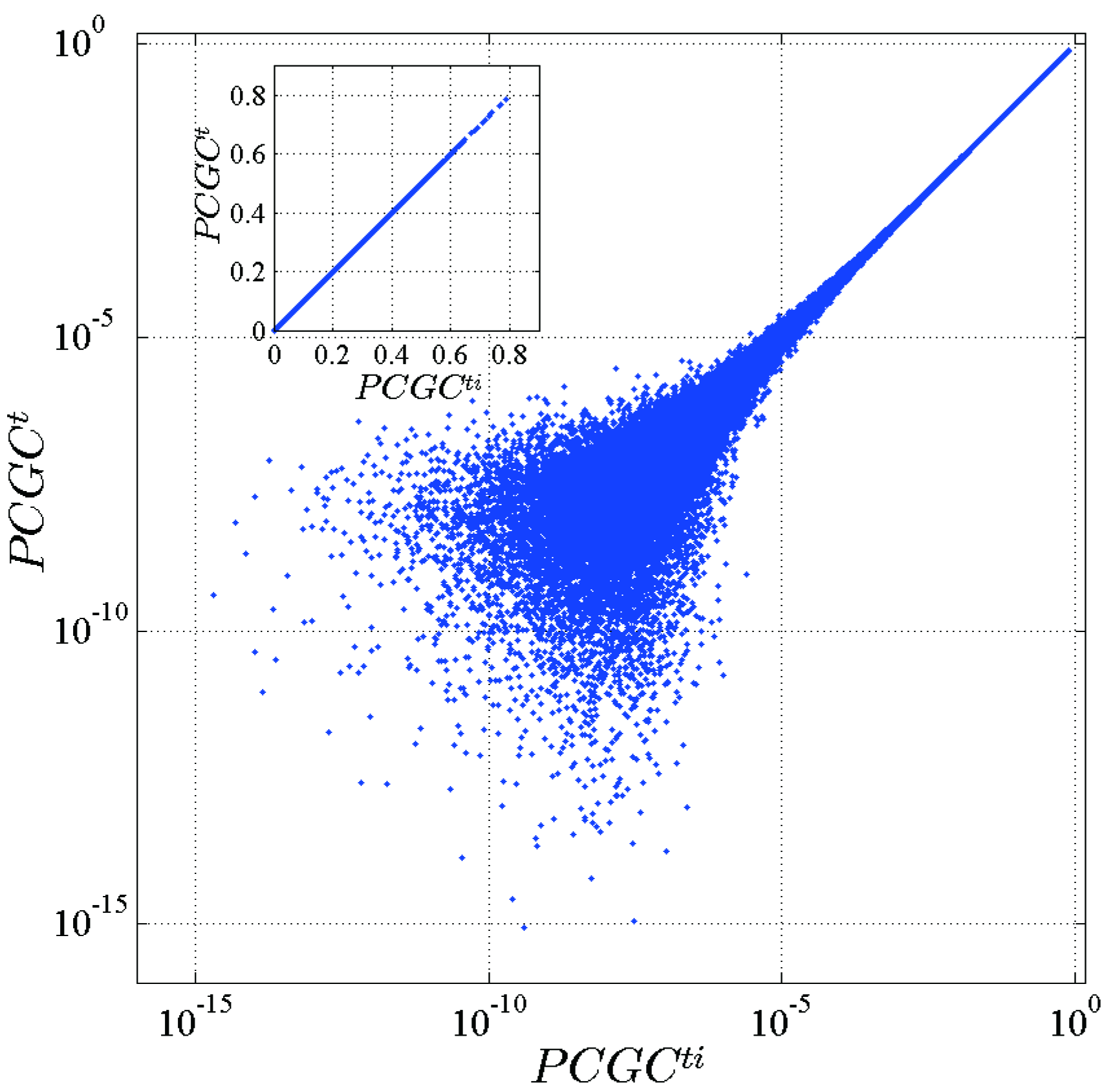

Supplement: Figure S3 — Log-log plot of PCGC ti and PCGC t . Inset, linear plot. (TIF) [file pone.0073670.s003.tif]

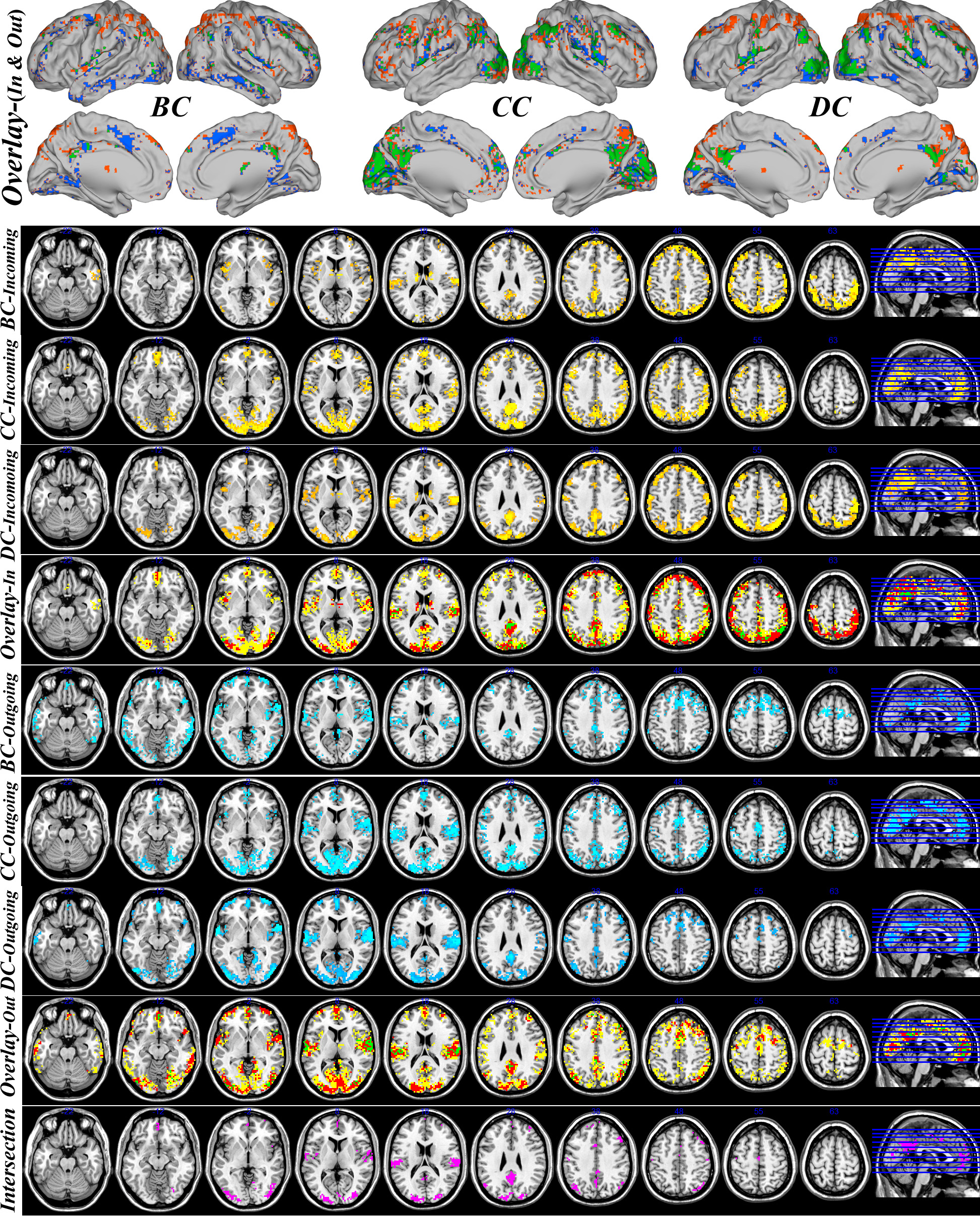

Supplement: Figure S4 — The spatial distribution of hub voxels of the weighted graph obtained keeping all the weights higher than a threshold of 0.3, with their value, and setting the rest to zero. In the top sagittal views, red indicates the incoming network hubs, blue the outgoing network hubs, while green the common hubs of incoming and outgoing network. Concerning the axial views, 1–3rd (5–7th) rows indicate the BC/CC/DC incoming network hubs. In 4th (8th) row, yellow indicates incoming (outgoing) regions which are hubs for one measure (hub-score of 1), red indicates incoming (outgoing) regions which are hubs for two measures (hub-score of 2), while green indicates regions which are hubs for all three measures (hub-score of 3). The last row indicates the regions that are at the same time hubs for incoming and outgoing network with hub score of at least 2. (TIF) [file pone.0073670.s004.tif]

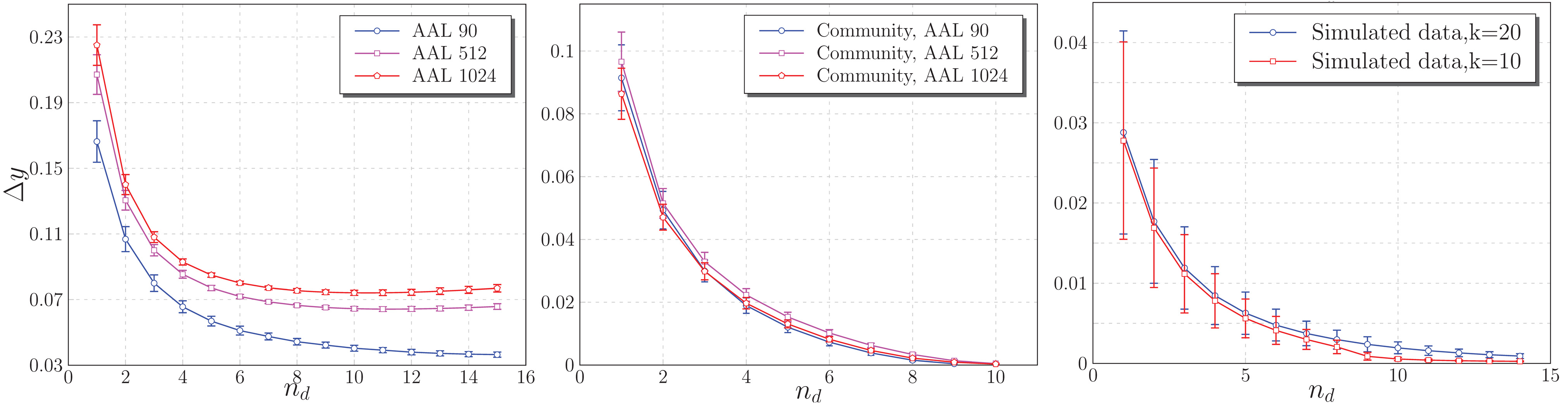

Supplement: Figure S5 — The mutual information gain (Δ y ), when the ( nd +1)-th variable is included, is plotted versus nd . The information gain is averaged over all the variables. Left: the conditioning set is calculated from the raw signal extracted from AAL-90/512/1024 template; Top right: is calculated on the signal extracted from each community; Right: curves for the simulated dataset; (TIF) [file pone.0073670.s005.tif]

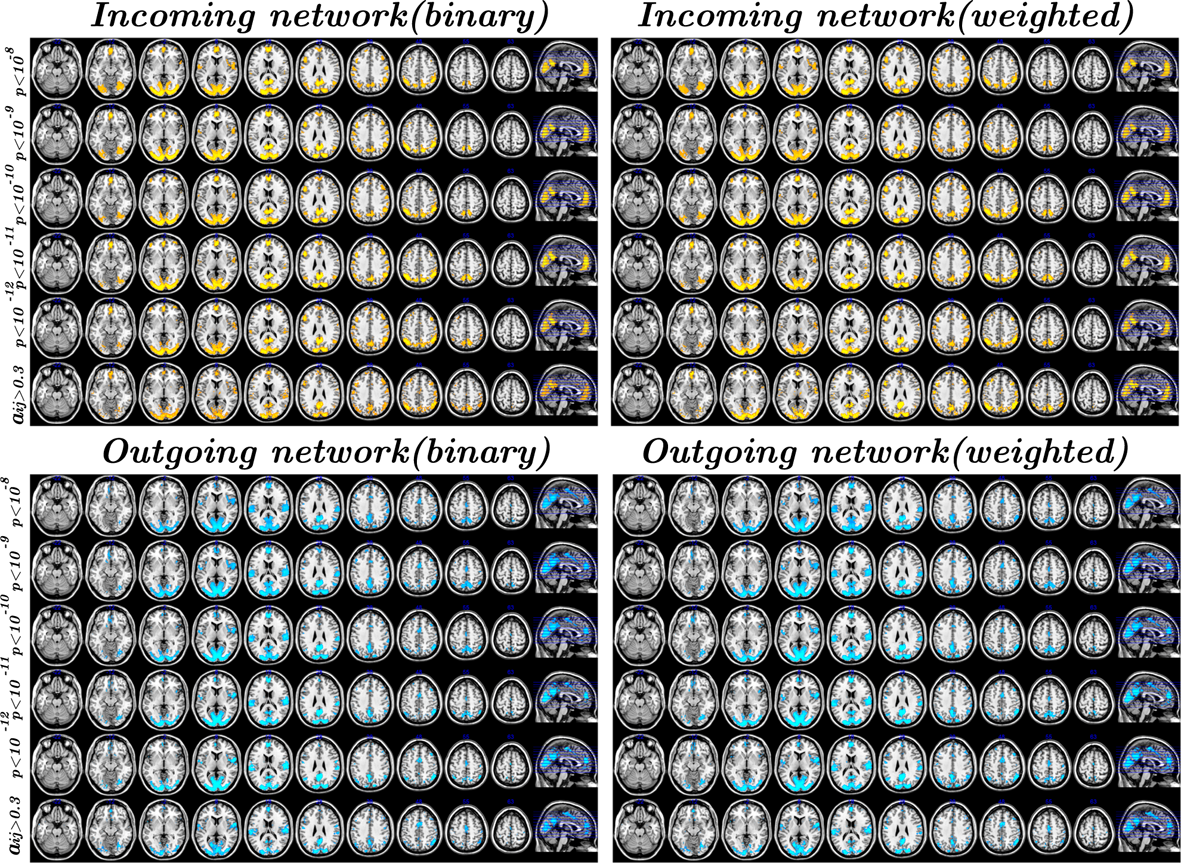

Supplement: Figure S6 — CC hubs distribution under different thresholds (rows from top to down, , , , , , aij >0.3 ). Top left, Incoming network (binary graph) CC hubs; Top right, Incoming network (weighted graph) CC hubs; Bottom left, Outgoing network (binary graph) CC hubs; Bottom right, Outgoing network (weighted graph) CC hubs. (TIF) [file pone.0073670.s006.tif]

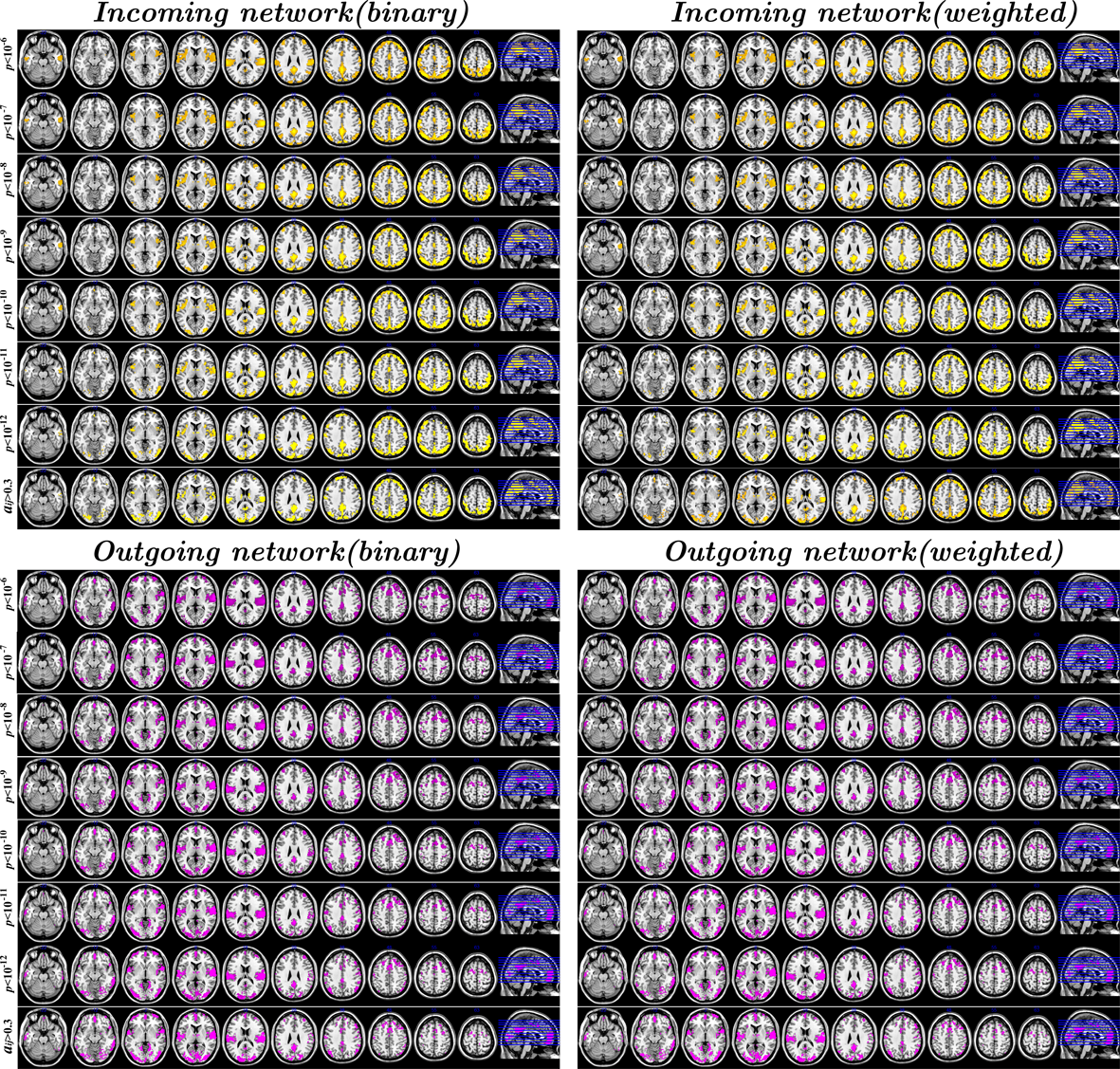

Supplement: Figure S7 — DC hubs distribution under different thresholds (rows from top to down, , , , , , , , aij >0.3 ). Top left, Incoming network (binary graph) DC hubs; Top right, Incoming network (weighted graph) DC hubs; Bottom left, Outgoing network (binary graph) DC hubs; Bottom right, Outgoing network (weighted graph) DC hubs. (TIF) [file pone.0073670.s007.tif]
